# Hospital Meetings, &c.

**Published:** 1900-04-07

**Authors:** 


### HOSPITAL MEETINGS, &C.

### CITY ORTHOPÆDIC.

The forty-ninth annual meeting of the City Orthopædic Hospital was held at Hatton Garden on the 28th ult., Mr. Walter Abbott presiding. From the accounts presented it appeared that the ordinary income for the past year amounted to £2,563, and the ordinary expenditure to £1,610. Donations of £69 for the extension fund were credited to extraordinary income, while the extraordinary expenditure for building improvements amounted to £1,684. Mr. Noble Smith (the hon. secretary) read the report. The committee expressed their satisfaction that the institution was now again in full working order after the reconstruction forced upon them on account of the insanitary condition of the buildings. The special appeal for the purpose was generously responded to, but a deficit of £3,400 still remained on the cost of the work absolutely necessary to be done. Much delay was occasioned by strikes and other causes, so that it was not until July 5th that the reopening took place, at which the Duke of Cambridge presided. Notwithstanding the closing of the hospital, it was found possible with great effort and forbearance on the part of the medical staff and other officials, to keep open the out-patient department during the whole period of reconstruction. The number of out-patients during the year was 2,242, an increase of 111 over those of the previous year. During the six months the wards were open the in-patients numbered 131. The committee pointed out that the increase in annual subscriptions was due to one of £250 from the Prince of Wales's Fund, given on condition that the two recently-constructed wards be filled and kept so. An alteration in one of the rules was assented to, the committee was re-elected, and the proceedings terminated.

### NORTH LONDON HOSPITAL FOR CONSUMPTION.

The annual Court of Governors and subscribers to the North London Hospital for Consumption, Mount Vernon, Hampstead, was held on Wednesday, March 28th, in the boardroom of the offices of the institution, 41, Fitzroy Square, W., Mr. F. D. Mocatta presiding.

The fortieth annual report of the board of management, which was presented to the meeting, recorded a remarkable improvement in the financial position of the hospital, and also the success of the "open-air" treatment. The committee stated that they had spared no effort to make the institution one of the most useful of the consumption hospitals, and they believed it would compare favourably with any other of its kind. The number of applications for admission had enormously increased during the past year. As the committee required £7,000 annually, of which the annual subscriptions totalled only £3,000, they pointed out that it was necessary to increase this source of income by at least another £3,000. Donations during the year had amounted to £1,966, and legacies received totalled £1,425. Whilst the liberal diet forming part of the "open-air" treatment had increased the expenditure, the cost of administration had been again reduced. As the result mainly of the success of the festival dinner held in May last, sufficient money was raised to pay off the mortgages on the property of the hospital at Hampstead, special thanks being accorded to Sir Henry Harben for largely contributing to this result. Mr. C. D. Rudd had made an annua contribution of £300 towards the cost of carrying out the new method of treatment, and all the 80 beds of the hospital were now open for the reception of patients. The average period of residence of patients had risen to 52 days, and the average number of beds occupied had increased from 52 to 72. The two wards set apart for the "open-air" treatment have been found insufficient, and it had been decided to erect a specially-designed wing for 40 beds, which it was hoped would be opened in May. The cost of the structure was £3,000, and is to form part of the final wing of the hospital. The medical report stated that the results under the new treatment had fully justified its adoption, and a comparison between an equal number of cases treated under the old and new systems showed that the latter was far superior. It is shown in tabulated statements that whilst in 168 cases under the old system only 34·1 per cent. showed improvement, under the "open-air" treatment the same number of cases gave an improvement of 76·1 per cent. The accounts showed that the total ordinary income for the year was £5,650, and the extraordinary (legacies) £1,425; whilst the total ordinary expenditure had amounted to £6,153, and the extraordinary expenses to £432, giving a credit balance of £489. In moving the adoption of the report, Mr. B. A. Lyon, chairman of the committee of management, explained the proposals with regard to the hospital extension now in hand, and instanced remarkable cures under the new treatment, which he said pleased and astonished those acquainted with the results and encouraged them to extend the principle as they were doing. Mr. Mocatta, in seconding the resolution, congratulated the subscribers on the results shown, which had led to the material support of the hospital and which would still more aid it. The report having been adopted, a resolution was approved revising the bye-laws. Some other formal resolutions having been agreed to, the proceedings terminated.

### THE MEDICAL GRADUATES' COLLEGE AND POLYCLINIC.

The first annual meeting of this institution was held at the College, on March 28th. The report showed that steady progress had been made in the amount of work done and especially in the number of attendances at the consultations and clinical lectures. There is no doubt that, judged by the balance-sheet, the finances are not in as flourishing a condition as might be wished, but we believe that the council fully recognise that such an institution as this, with its large range of work, cannot at once become financially prosperous, and that they have full confidence that by carrying out their programme and affording a centre at which post graduate work shall always be going on, at which post graduate classes on special subjects shall be held, and at which clinical lectures, demonstrations, and consultations shall be open to the members, day by day, almost the year round, a great impetus will be given to post graduate study in London, and that ultimately financial success will be assured. A good deal was said at the meeting about the desirability of the general practitioners being more fully represented on the council, and in regard to this, Sir William Broadbent assured the members that proper arrangements would be made when that body was next elected. Mr. Hutchinson, in a cheery and hopeful speech, said that the pecuniary liabilities of the Polyclinic ought not to be entirely thrown upon the medical profession. Its work, he said, was not solely for the benefit of medical men, its aims were for the benefit of the community, and he thought it had a bona-fide claim for pecuniary aid. With much of this we can cordially agree, and it is to be hoped that some rich people will come forward to assist the charity side of the institution, that, namely, which is concerned with the provision of free consultations in obscure or interesting cases for such as are unable to pay an ordinary consultation fee. This is really a form of hospital work which may be supported by those who regard hospitals as having a special claim upon their purses. Sir William Kynsey moved and Dr. Ewart seconded the re-election of the council, and this was carried.

### THE WEST END HOSPITAL FOR EPILEPSY AND PARALYSIS.

The twenty-first annual meeting of the West End Hospital for Epilepsy and Paralysis was held at the institution, 73, Welbeck Street, on the 23rd ult., Mr. G. E. Porter, the chairman, presiding. Dr. Wood read the medical report, from which it appeared that the number of new in-patients during the past year was 325. The out-patients numbered 3,480, The number of massage treatments was 7,020 ; the electrical, 11,264. The total out-patient attendances were £33,971. The beds were all full, and there was a long list of the names of children waiting admission. A new operating room was badly needed. The financial statement gave the ordinary receipts as £3,784; ordinary expenditure, £3,241. The extraordinary expenditure was £315, but there were no extraordinary receipts. The sum due by the building to the general fund was £6,745, as compared with £6,430 a year ago. Attention was called to the fact that the grants from the Hospital Saturday and Sunday Funds had both been increased, the figures being £87 and £125, as compared with £76 and £82 last year. £100 had been received from the Prince of Wales's Fund towards the expense of the new operating room. Although many valued subscribers had been removed by death, yet the annual subscriptions again showed such an increase as indicated the confidence and interest of the public in the work of the hospital. The Chairman, in moving the adoption of the report, which was agreed to, also referred to the need for additional sleeping accommodation for the nursing staff, some of whom had to be boarded out, an arrangement which entailed much expense and was inconvenient in many ways. He appealed confidently for funds to carry out the improvements required to enable the hospital to extend its sphere of usefulness. General Mercier said he thought the increase of their subscriptions was largely due to their hon. treasurer, Captain Dowell, and his system of appeals. Votes of thanks closed the proceedings.

### POPLAR HOSPITAL.

Quite a jubilant note was struck in the report presented by the committee to the annual meeting of the Poplar Hospital for Accidents on the 20th ult., at which the Hon. Sydney Holland, the chairman, presided. Eighteen hundred and ninety-nine would ever be famous, they declared, in the annals of the hospital, on account of the magnificent generosity of the Drapers' Company. Realising that the London Hospital was the only adult medical hospital for the whole of East London, that company had presented the Poplar Hospital with £4,200 to buy the site, had promised £10,000 towards the building, and £1,000 a year towards the upkeep of an addition to the hospital of wards containing twenty medical beds. This was equivalent to a gift of £45,000. By the summer of 1901 they hoped to see the "Drapers' Wing" in full working order. Plans had been obtained, and the contract signed for £13,683. This would give three wards of ten beds each, but only two wards would be opened at first. Over and above the noble contribution of the Drapers' Company, the cost with furnishing would amount to £5,000, and to defray this all friends of the hospital were appealed to. But apart from the generous donation of the Drapers' Company, the past year had been a good one, both in receipts and in work done. They had received no less than £15,231 (of which the ordinary income was £8,868, and legacies £2,141, plus the £4,200 for the purchase of the site), while the expenditure had only been £11,821 (ordinary, £6,404), and had, therefore, been able to add £3,400 to reserve. The in-patients had numbered 877, as compared with 993 in 1898; but the average number of beds occupied daily in 1899 was 56, as against 51 in 1898—itself the record year of work for 44 years. As instancing the use for the hospital, it was mentioned that there were at the moment in one ward three poor fellows with broken backs. The report referred in most cordial terms to Miss Bland (the matron) and the nursing staff. Miss Bland and Miss Smith (the home sister) had that year both qualified in massage, so that all the nurses could now be trained, to the great benefit of many patients who needed that treatment. Mr. Openshaw and Mr. Stonham had both gone to the front, attached to the Yeomanry Field Hospital. Mr. Hugh Rigby had been appointed to do their work on the honorary staff, in conjunction with Mr. Roxburgh. Mr. C. B. Howse, the senior resident surgeon, had also received an appointment with the forces in South Africa, having been gazetted to the 11th Battalion Imperial Yeomanry as surgeon, with the rank of captain. Thanks were again expressed to Mr. Edmund Hughes, the managing director of the Tilbury Lighterage Company, who had again arranged for weekly trips for the nurses down the river in one of the company's tugs—a way of helping them than which it would be difficult to conceive a kinder.

The Chairman moved the adoption of the report, goin over it and emphasising its points. They had not, he said, had to turn away a single case of accident during the year; they kept their expenditure down, but they treated their nurses decently. They were always willing to work longer and harder than they asked them to, but he thought they had no right to economise at the expense of their nurses and at the sacrifice of their happiness in life as they got older. That had been a record year for help from the working classes, and in this respect that hospital beat any other in London. The motion was seconded and adopted, and a heartily applauded vote of thanks to the chairman ended the meeting, which was as usual well attended by the working-men supporters of the institution.

### ROYAL SEA-BATHING HOSPITAL.

The 109th report of the Court of Directors was presented at the meeting of the Royal Sea-Bathing Hospital, Margate (formerly known as the Royal Sea-Bathing Infirmary), held at the offices, Charing Cross, on Monday, 26th ult., Mr. Michael Biddulph, M.P., the treasurer, presiding. The financial statement showed the total ordinary income for the year to have been £5,773, and the ordinary expenditure, £7,501. The extraordinary income was—legacies, £17,252; special appeal, £614; together, £17,866. The extraordinary expenditure (building and repairs) was £489. It was decided in March last to enlarge the scope of the charity, and twelve beds were set aside for cases of a non-tubercular character. This provision had been found most useful, cases that would otherwise have been excluded having been treated with marked success. The average cost per bed occupied was £1 6s. 11½d.; 487 patients were discharged during the year, of whom 12 per cent. had recovered, 48 per cent. were greatly benefitted, and only 1 per cent. had died. The report called renewed attention to the fact that the open-air treatment of tubercular disease had in various particulars been carried out at that hospital for many years. From the resident surgeon's report it appeared that the main features of the treatment adopted were: Liberal diet and abundance of fresh air; cod liver oil and tonics; sea bathing, and surgery. The beds were so constructed that they could be easily wheeled on to the verandahs, and, with few exceptions, every patient spent a considerable part of the day in the open air. The verandahs being roofed with glass, enabled this to be done in any weather, winter and summer. In the summer months those patients whose physical condition allowed of it bathed in the sea; salt water was, however, laid on in the bath-rooms, enabling the treatment to be employed all the year round. Two hundred and fifty-three operations, necessitating the administration of anæsthetic, had been performed. In moving the adoption of the report, the Chairman said that during the year they had been renovating the hospital, which was now in most admirable condition. They had increased the staff, and generally were on a better footing than ever before. They had had two good legacies, one of £10,000 and one of £5,000, and although obliged to spend a part of these on the renewals, they had been able to invest a considerable portion. The report was adopted, after which Lord Derby was re-elected president, as were also the vice-presidents and other officers. The meeting having been made special, alterations in the laws were carried by which subscribers will be able to send six patients for a month instead of two for three months. The effect of this is to allow of patients not found to be benefitting to be sent away at the end of a month without a feeling of grievance that they have not been allowed the time to which they are entitled, as has sometimes been the case. The proceedings then terminated.

### HOME HOSPITALS ASSOCIATION.

The Duke of Northumberland (the president) took the chair at the twenty-second annual meeting of the Home Hospitals Association (for paying patients) at Fitzroy House, on the 28th ultimo. In moving the adoption of the report, his Grace said he could not help recalling the fact that it was just twenty years since they first occupied that house, and nineteen years since last he had the honour of presiding at one of those meetings. The progress the society had made in that time had been very great. In 1880 they had 15 beds; now they had 24. In 1880 they had 118 patients; in 1899, 292. In 1880 their income was £2,136; in 1898, £5,326. In 1880 the expenditure was £2,102; in 1898 £5,365. He thought those figures spoke volumes; but that was not all. The institution of the Nurses' Home was a very important step and had had very important results. They might also congratulate themselves on the circumstance that, whereas twenty years ago theirs was the only, or almost the only, institution of its kind, now their example had been taken up, and such hospitals were by no means rare. They had led the van, and he trusted that for many years they would maintain their position. Their object was a very large one, covering a considerable area, because there were very many people between the very poor and the very rich to whom the Home Hospital was of the greatest benefit —that class which, with limited means, was accustomed to a certain amount of comfort when in health, and was yet quite unable to get the quiet, the nursing, the food, and so forth so necessary to the welfare of a sick person. That their work was approved was proved by the fact that every year numbers of people were obliged to go elsewhere because they could not accommodate them. He trusted the day would come when such institutions would be so numerous that all who needed them would be able to be taken in. It was to be hoped also that the principle of people occupying hospitals paying for part of the cost of their treatment might be carried further. They all felt how good was the work carried on by the great London hospitals, but there were many who ought not to take advantage of these charities. No doubt the poorer classes did do a great deal towards the support of the hospitals, but he was not sure that the sick people themselves, and their relatives, did all they might in the way of contributing towards the benefits they received.

The report, which was then adopted, stated that during the past year 400 applications for admission were received. Of these 332 were admitted, 40 being friends of patients. Of those not admitted 55 were refused for want of room, 6 were unsuitable, and in three cases the terms were beyond the patients' means. The average number of cases throughout the year was 15, and the average length of stay was 12 days. Sixty-two medical practitioners in London had had patients in the hospital during the year, an increase of 4 over 1898. Owing to extensive repairs in the basement of No. 16, Fitzroy Square, it was found advisable to close that house for 38 days in the autumn, but notwithstanding this the number of patients admitted during the year was only 21 less than in 1898. The total receipts had also necessarily decreased, but the financial condition of the association was satisfactory, and compared favourably with that of preceding years. The medical board of reference had been strengthened by the election of Sir William MacCormac, Mr. Nettleship, Mr. A. Barker, and Mr. Bilton Pollard. Messrs. Barker and Nettleship have also been appointed representatives of the medical board on the committee of management. The chief change in the staff of the hospital was the resignation of Miss Winwood, the assistant superintendent, who had been succeeded by Miss Roberts. Mr. Turnbull, the solicitor to the association, had retired through ill-health, and Messrs. Fladgate and Co. had been appointed in his place. Great regret was expressed at the death of Mr. H. F. Cox, a most regular attendant on the Committee of Management.

Colonel Hamilton proposed the re-election of the officers and the committee, and in seconding the motion, which was carried with acclamation, Mr. Freeman gave some personal reminiscences of his experiences of that day, 20 years ago, when the hospital was first opened. He recorded his warm appreciation of the nursing staff of that time, and congratulated the present nurses on their work and appearance.

Mr. Bland Sutton brought forward, for the consideration of the committee, the suggestion, in view of the advances made in medical and surgical science during the 20 years since they had occupied those buildings, and the pressing need for an operating theatre, that they should erect on the whole of the ground at their disposal a building worthy of their work, and which would give them the increased accommodation so much needed.

Mr. Cox, as treasurer, said the one grave objection to such a scheme was the absence of money to carry it out. It would need something like £20,000, and he doubted if they could get funds from the public for an object not entirely charitable, for though they had never made any money yet they were on a business footing. It was a charming and a pleasing scheme, which he admitted would give them double the space for beds, and be advantageous in other ways, but he did not see how it was to be carried out.

Mr. Bland Sutton said possibly a limited company might be formed for the purpose, and he was sure there were many who would be glad to subscribe to it. His views were those of others of the medical staff.

The Chairman said the suggestion was a useful one, but the subject was of course too big for discussion there and then. He was sure the committee would give it their consideration.

Votes of thanks terminated the proceedings.

### ST. PETER'S HOSPITAL.

Mr. Bevan, the treasurer, took the chair at the thirty-second annual meeting of St. Peter's Hospital for Stone, Henrietta Street, on Tuesday, March 27th. He moved the adoption of the report, and congratulated the governors and subscribers on the success of the hospital in so far as its work was concerned, though their balance-sheet showed a rather serious state of things. It was gratifying to note that the number of patients continued to increase. The in-patients in the past year numbered 562, as compared with 502 in 1898. Almost all their beds were constantly occupied; at that moment 26 out of their 27 were full. But not only had they more patients but they were getting greater benefit. The mortality from operations continued to decrease. In their first decade, 1874 to 1883, the percentage was 15·25; from 1884 to 1893 it had fallen to 8·29; and in the six years 1894 to 1899 it had come down to 4·55, last year's being actually 4·41. This, he thought, reflected the highest credit on their surgeons, and showed that the greatest care was taken of the patients. Even in the case of the few who did die, many of them if they had come earlier might have lived much longer. As it was, of the 562 in-patients only 19 died, and some of these were practically moribund on arrival. They made a point of not refusing such cases, as some hospitals did, but did their best to relieve them so far as possible. In the out-patients' department there had been a slight decrease, the numbers being 4,435, and the attendances registered 37,474. The falling-off might be attributed, perhaps, to the war—soldiers who used to come to them being now away—but probably more to the dislocation occasioned by the improvements that had been carried out in that department, which had been entirely remodelled and provided with new electrical and surgical appliances. These alterations had cost about £600. When he turned from the surgical aspect to the financial he was sorry to say it did not present so cheerful an aspect. Their income was £3,396, and their expenditure £4,540, of which £857 was for building and the out-patient department improvements. There was thus a deficit on the year of £1,144, and they had been obliged to sell out some of their stock. The way out of the difficulty seemed to be a special appeal, but the present was not a good year for that.

Mr. Christy seconded the motion, observing that the outlay in the out-patients' department enabled the surgeons to do better work than hitherto. He commented on the fact that a large number of advanced men attended the practice of the hospital, and were always welcomed by the staff, especially at operations. They were thus doing public good, and a high opinion was frequently expressed in the press, and especially in the foreign press, of the work being done at the hospital.

Mr. Hurry Fenwick moved the adoption of the medical report, which was carried, as were cordial votes of thanks to the staff and to the chairman.

